# Quantification of Removable Prosthesis Plaque Area Coverage Among Adult Patients: A Systematic Review and Meta‐Analysis

**DOI:** 10.1002/cre2.70319

**Published:** 2026-02-25

**Authors:** Tong Wah Lim, Maxstein M. Abuzaid, Jade Yuen Kei Wong, Kar Yan Li, Michael Francis Burrow, Colman McGrath

**Affiliations:** ^1^ Division of Restorative Dental Sciences, Faculty of Dentistry The University of Hong Kong Hong Kong SAR China; ^2^ Dental Public Health, Faculty of Dentistry The University of Hong Kong Hong Kong SAR China; ^3^ Division of Applied Oral Sciences and Community Dental Care, Faculty of Dentistry The University of Hong Kong Hong Kong SAR China

## Abstract

**Objective:**

To investigate removable dental prosthesis plaque area coverage assessment methods and conduct a meta‐analysis of prosthesis cleanliness status of adult patients wearing removable dental prostheses, quantified by using computerized planimetric assessments.

**Materials and Methods:**

The present study was registered with PROSPERO and conducted following the principles published by the working group of the Joanna Briggs Institute (JBI). Literature searches were conducted across PubMed, Ovid MEDLINE, Embase, Cochrane Central Register of Controlled Trials, and Scopus. Quality assessments were performed according to a JBI critical appraisal checklist across nine criteria. The results were pooled using a frequentist random effects meta‐analysis to estimate the event proportion. Sensitivity analysis was performed by excluding some studies.

**Results:**

Initially, 6342 articles were identified. After screening titles and abstracts, 141 studies remained. Ultimately, 57 studies were included in this systematic review. Various visual indices were employed in clinical studies for quantifying dental prosthesis plaque area. A meta‐analysis was performed on 12 computerized planimetric assessment studies. The estimated pooled percentage plaque coverage area, as measured using the computerized planimetric assessments, was 37.06% (95% CI: 25.89%–48.23%). The heterogeneity in the meta‐analysis was “considerable.” In a subgroup analysis according to the software used, the heterogeneity within the Adobe Photoshop group was much reduced, *I* ^2^ = 0% (*p* = 0.734).

**Conclusions:**

The existing evidence demonstrated poor removable prosthesis cleanliness among adult patients. Furthermore, there is a lack of standardized plaque quantification methods for both research and clinical applications, with most studies relying on visual indices for plaque assessment.

## 1 Introduction

Removable dental prostheses remain a prevalent treatment option for tooth loss worldwide. Despite the decreasing prevalence of total tooth loss among adults, demographic changes have led to consistent and even slightly increasing demand for removable prosthesis services (Steele et al. 2019). Although dental practitioners can provide a remarkable range of treatments for preserving and restoring natural dentition, there continues to be a widespread need for removable dental prosthesis care, particularly among older adults and patients with lower socio‐economic status. Various factors contribute to the tendency of prosthesis surfaces to plaque deposition. Irregularities and roughness of prosthesis materials create niches for biofilm adherence, particularly on the fitting surface, which can be challenging to clean mechanically and chemically (Coulthwaite and Verran 2007). In addition, the deposition of food debris, reduced saliva flow, and compromised immune response in older adults wearing removable prostheses can further exacerbate biofilm deposition (Tada and Hanada 2010).

Prosthesis plaque has essentially a similar structure and composition as dental plaque formed on natural teeth, but with an increased number of Candida spp (Coulthwaite and Verran 2007; Redfern et al. 2022). Numerous studies have also demonstrated the high prevalence of pathogenic bacteria, particularly respiratory pathogens, residing on prosthesis surfaces (Coulthwaite and Verran 2007; Lim, Huang, Jiang, et al. 2024; Lim, Huang, Zhang, et al. 2024; Lim et al. 2024a; Sumi et al. 2003, 2002). Associations or mutualistic interactions between prosthesis plaque and oral and systemic diseases in the absence of optimal oral hygiene measures were reported, particularly in high‐risk patients (Coulthwaite and Verran 2007; Ewan et al. 2015). However, prosthesis hygiene in the community is seldom assessed as part of routine oral examinations, unlike the Basic Periodontal Examination, and plaque and bleeding scores. A major concern is that previous studies have reported poor prosthesis hygiene status among adult patients, particularly among older adults in long‐term care facilities (Mylonas et al. 2014; Lim, Lee, et al. 2025). Possibly, the lack of standardized removable prosthesis hygiene education and patient awareness contributes to unsatisfactory prosthesis cleanliness in the community (Mylonas et al. 2014).

Quantification of removable prosthesis plaque offered crucial insights into health conditions related to prosthesis plaque, has been able to investigate prosthesis hygiene in the community, and also examine the effectiveness of prosthesis hygiene interventions (Lim, Lee, et al. 2025; Knezović Zlatarić et al. 2002; Ng et al. 2021). Various techniques for quantifying prosthesis plaque have been developed, including measurements of dry or wet weight, analysis of oxygen usage, conducting biochemical tests, counting the abundance of bacteria or fungi, and employing visual indices or planimetric assessments (Thu et al. 2024). The wide range of assessments and lack of standardization across all methods further complicates the process of comparing the effectiveness of different prosthesis hygiene care products. Among them, visual indices and planimetric assessments were commonly adopted for clinical studies (Paranhos and Silva 2004; Coulthwaite and Verran 2009).

The majority of clinical visual plaque indices produce ordinal scale scores rapidly, which can be beneficial for epidemiological or audit studies (Mylonas et al. 2014). However, they rely on an experienced examiner's subjective evaluation, potentially leading to biases, reduced reproducibility across studies, and lower accuracy and sensitivity compared with computerized planimetric assessments. This ultimately generates random and systematic errors (Browner et al. 2022). Previous studies have reported that planimetric plaque evaluation using computer software is more dependable and objective than traditional visual plaque indices in research studies (Lim, Pan, et al. 2023). This method provides linear measurements, improved reproducibility, higher objectivity, and enhanced discriminatory capabilities (Coulthwaite and Verran 2009; Lim, Pan, et al. 2023; Söder et al. 1993; Smith et al. 2001). In addition, it demonstrates a strong correlation with prosthesis cleanliness, plaque weight, viable microbial counts, and visual assessment (Lim, Huang, Jiang, et al. 2024; Lim, Huang, Zhang, et al. 2024). The development of automated systems for plaque image analysis diminishes investigator involvement and subjectivity (Lim, Pan, et al. 2023; Del Rey et al. 2023).

At present, there is no gold standard for removable prosthesis hygiene assessment, nor is there a widely adopted method for plaque area quantification. In addition, to achieve optimal oral and systemic health, the removable prosthesis cleanliness status in the prosthesis‐wearers community should be investigated and regularly monitored, potentially informing patients on the best prosthesis hygiene care. Therefore, this review aimed to examine existing literature and conduct a systematic review of prosthesis plaque area coverage assessment methods, as well as a meta‐analysis of the prosthesis cleanliness status of adult patients wearing removable dental prostheses, as quantified by using various computerized planimetric assessments.

## 2 Materials and Methods

### 2.1 Eligibility Criteria, Search Strategy, and Protocol Registration

In light of previously published literature concerning the methodologies that have been employed to assess the extent of removable prosthesis plaque coverage, the subsequent inquiry “What is the plaque area coverage in removable dental prostheses for adult patients within the community?” has been devised using the condition, context, and population (CoCoPop) mnemonic provided by the Joanna Briggs Institute (JBI) (Munn et al. 2015). The inclusion and exclusion criteria were determined following the CoCoPop framework, as outlined below:

i. Condition (Co) refers to the plaque formed on the removable prosthesis surfaces that were quantified by visual assessment indices or planimetric assessments, including paper‐weighting, point counting, or computer software. However, prosthesis cleanliness after any type of intervention was excluded.
ii. Context (Co) pertains exclusively to clinical studies that investigated removable prosthesis cleanliness, regardless of the type of study design. However, literature and systematic reviews, in vitro studies, protocols, ongoing trials, case reports, and abstracts were excluded.
iii. Population (Pop) encompasses adult patients wearing removable dental prostheses (implant‐retained prostheses, complete removable prostheses, partial removable prostheses, tooth‐supported overdentures), regardless of the quality of prostheses and the patient's health condition.

This review was registered with PROSPERO, The International Prospective Register of Systematic Reviews (Ref: CRD42023489394), published on December 15, 2023, and adhered to the guidelines established by the working group of the JBI for appraising systematic reviews of prevalence (Munn et al. 2015).

### 2.2 Literature Search and Study Selection

The search strategy is presented in Appendix 1, which was verified by a dental and medical librarian of the Faculty of Dentistry at the University of Hong Kong. A comprehensive literature search was conducted across five online databases, namely PubMed, Ovid MEDLINE, Embase, Cochrane Central Register of Controlled Trials (CENTRAL), and Scopus, with the most recent search performed from October 27 to October 30, 2023. The search was restricted to English‐language publications, but there was no restriction on the year of publication. Removal of duplicated articles was performed by using EndNote X9 software (Thomson Reuters, Philadelphia, PA, USA). Two independent reviewers (M.M.A. and J.Y.K.W.) conducted the search by reviewing titles and abstracts in accordance with the eligibility criteria. Subsequently, a full‐text analysis was performed on shortlisted studies. The reviewers compared the included studies and resolved any discrepancies through mutual discussion or consulted a third reviewer (T.W.L.).

### 2.3 Data Collection and Effect Measures

The primary focus was on obtaining the prosthesis plaque area coverage (mean, standard deviation, and proportion) present on removable prostheses, as reported or calculated from included studies. No limitations were imposed on the type of removable prosthesis, plaque quantification methods, participants’ health status, or data source, except for findings of prosthesis cleanliness following any intervention. Studies encompassed removable prosthesis plaque area coverage or indices in patients with healthy or diseased mucosa. Data from control groups not subjected to any intervention were also considered for inclusion. The collected data were compiled into a standardized spreadsheet. The extracted information included the name of the study, authors’ names, year of publication, location of the study, the aim of the study, study type, sample size, participants’ details (sex, age range), removable prosthesis characteristics (design, year of usage, and number and type of surfaces assessed) type of disclosing agent, assessment methods (visual indices or planimetric assessment methods including paper‐weighting, point counting, or computer software), name of the method/software used for assessment, and outcome measures (total number of prostheses included, percentage or mean with standard deviation of plaque coverage for visual indices, mean percentage and standard deviation of plaque coverage for planimetric assessment methods, standard error, and confidence intervals if available). The reviewers compared their findings and resolved any discrepancies through mutual discussion. The data were subsequently utilized for meta‐analysis.

### 2.4 Assessment of Methodological Quality and Risk of Bias

The methodological quality and risk of bias of all included studies were evaluated by two independent reviewers (J.Y.K.W. and M.M.A.). In cases where disagreements arose between the reviewers, resolution was achieved through discussion and, if necessary, the involvement of a third reviewer (T.W.L.). A revised validated tool, the JBI critical appraisal checklist (Appendix 2) was adopted in this review. This checklist consisted of nine criteria, including the appropriateness of the research design, sample size, study setting, data analysis, diagnostic criteria, methods of identification, and statistical analysis.

### 2.5 Statistical Analysis

The findings from the included studies were synthesized in a narrative format, considering all characteristics of the studies. The results were then pooled using a frequentist random effects meta‐analysis to estimate the event proportion. To achieve this, the calculation based on the normal approximation to the binomial distribution was applied to the effect estimate and the corresponding standard error in proportion, followed by using the Procedure metan in Stata 16.0 (StataCorp. 2019. Stata Statistical Software: Release 16. College Station, TX: StataCorp LLC.). The pooled event prevalence or proportion and the corresponding 95% confidence intervals were estimated by random‐effect meta‐analysis. Heterogeneity was evaluated using the *I* ^2^ statistic, with 0% to 40% indicating unimportant inconsistency, 30% to 60% indicating moderate heterogeneity, 50% to 90% indicating substantial heterogeneity, and >90% indicating considerable heterogeneity. Subgroup analysis was performed according to the computer software to investigate possible causes of heterogeneity among studies. When three or more studies were included in the meta‐analysis, prediction intervals were also presented to account for heterogeneity. Begg's test and Egger's test with funnel plot were used to assess the publication bias. The Begg test assessed if there was a significant correlation between the ranks of the effect estimates and the ranks of their variances. The Egger test used linear regression to assess the relation between the standardized effect estimates and the standard error. Sensitivity analysis was also performed by removing some studies that had extreme outlier estimates compared with others.

## 3 Results

**Figure 1:**
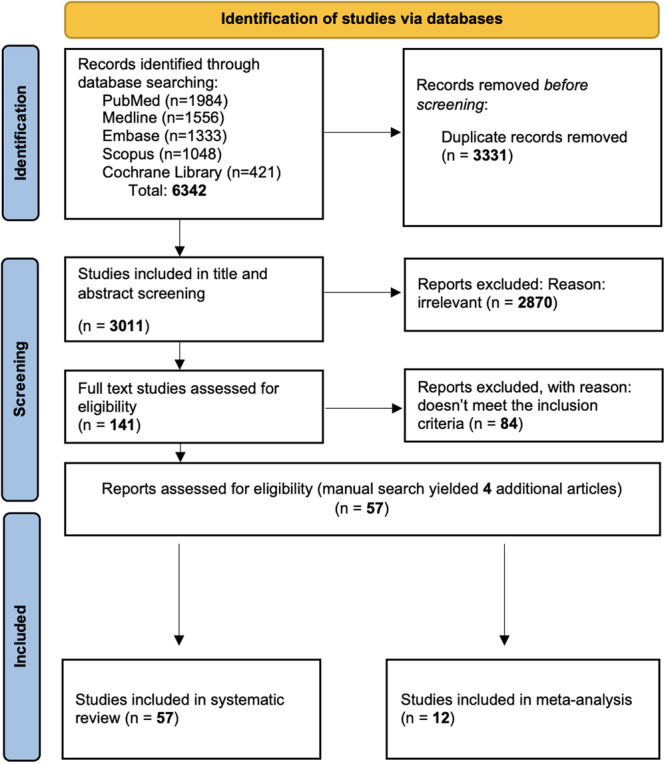
Flow diagram for selection of articles.

A total of 6342 studies were retrieved through the primary literature search as follows: 1984 studies from PubMed, 1556 studies from Medline, 1333 studies from Embase, 1048 studies from Scopus, and 421 studies from CENTRAL. The initial screening was done based on the title and abstract only, and 141 studies remained and underwent a full‐text review. Four additional studies were added through manual search, resulting in a total of 57 studies eligible for this systematic review, with a Cohen's kappa coefficient (κ) of 0.81 (Figure 1). Among them, two adopted both visual index and planimetric assessment. A summary of included studies is listed in Tables 1 and 2.

**Table 1:** Characteristics of included studies in the systematic review.

No	Study	Location of the study	Type of the removable prosthesis	Plaque assessment method	Index	Finding
1.	Muenchinger (1975)	Community, School of Dentistry, The University of Michigan, Ann Arbor, Michigan	Complete	Visual	Muenchinger (1975)	For group A (electronsonic), mean value was 15.38
2.	Budtz‐Jörgensen (1977)	Community, Department of Prosthetic Dentistry, Aarhus, Denmark	Complete	Visual	Budtz‐Jörgensen (1977)	15.79% scored 1, 36.84% scored 2, and 47.37% scored 3
3.	Ambjørnsen et al. (1984)	Community, University of Bergen, Bergen, Norway	Complete	Visual	Ambjørnsen et al. (1982)	The mean value of plaque index was 1.4 ± 0.78.
4.	Ambjørnsen and Rise (1985)	Community, Municipality near Oslo	Complete	Planimetry: point counting	Ambjørnsen et al. (1984)	The mean value of control group (preexperimental scores) was 46% (expressed as percentage)
5.	Badawi et al. (1986a)	Community, Faculty of Oral Dental Medicine, Cairo University	Complete	Visual	Badawi et al. (1986a)	Nonpolished (Group II) has 31.78% plaque coverage (percentage was used but not score)
6.	Badawi et al. (1986b)	Community, Faculty of Oral Dental Medicine, Cairo University	Complete	Visual	Badawi et al. (1986b)	Heat‐curing acrylic (group I) scored 63.75%, while cold‐curing acrylic (group II) scored 64.25% plaque coverage
7.	Budtz‐Jjörgensen and Kaaber (1986)	Community, Prosthetic treatment in the Department of Prosthetic Dentistry, Royal Dental College of Arhus,	Complete	Visual	Budtz‐Jørgensen et al. (1983)	There was a significantly reduced plaque on the glazed half of the denture
8.	Miyazaki et al. (1992)	Nursing homes, Institutions for the elderly in Kitakyushu, Japan	Complete and partial	Visual	Vigild's criteria	The mean value of the visual index for participants with good physical and mental condition was 1.06, while for fair condition was 1.2, and for poor condition was 1.29
9.	Jeganathan et al. (1996)	Community, Singapore	Complete	Visual	Jeganathan et al. (1996)	The mean value of visual index was 2.56 ± 1.28
10.	Keng and Lim (1996)	Community, National University of Singapore, Singapore	Complete	Visual	Quigley–Hein scale	The mean value of visual index was 5.78
11.	McCabe et al. (1996)	Community, Newcastle upon Tyne, United Kingdom	N/A	Visual	McCabe et al. (1996)	The mean value of visual index for control group was 4.65
12.	Russell et al. (1999)	Hospital setting, Goldwater Memorial Hospital, a 1000‐bed public hospital in New York City	N/A	Visual	Russell et al. (1999)	The mean value of visual index for dental outpatient (control group) was 0.3 ± 0.4
13.	Celić et al. (2001)	Community, University of Zagreb	Complete and partial	Visual	Tarbet index	For complete maxillary dentures, 27.3% scored 0, 25% scored 1, 28.4% scored 2, 11.3% scored 3%, and 8% scored 4
14.	Kulak‐Ozkan et al. (2002)	Community, Department of Prosthetic Dentistry, University of Marmara, Istanbul, Turkey	Complete	Visual	Budtz‐Jørgensen and Bertram (1970)	15.70% scored excellent, 35.70% scored fair, and 48.60% scored poor
15.	Knezović Zlatarić et al. (2002)	Community, Department of Removable Prosthodontics, School of Dental Medicine, University of Zagreb, Croatia	Partial	Visual	Tarbet index	45% scored 1
16.	Barbeau et al. (2003)	Community, Universite´ de Montre´al, Faculty of Dentistry, Canada	Complete	Visual	Jeganathan et al. (1996)	The mean plaque coverage percentage for healthy group was 32.93%
17.	De Visschere et al. (2006)	Nursing homes, Belgium	Complete and partial	Visual	Augsburger and Elahi (1982)	The mean value of plaque index for control group was 2.13 ± 0.88
18.	Peltola et al. (2007)	Hospital setting, Laakso Hospital, Helsinki, Finland	Complete and partial	Visual	Peltola et al. (2004)	30% scored poor, 51% scored moderate, and 19% scored good
19.	Al‐Dwairi (2008)	Community, Dental Health Centre of Jordan University of Science and Technology, Irbid‐Jordan	Complete	Visual	Ambjørnsen et al. (1982)	29% scored 0, 34% scored 1, 20.33% scored 2, and 16.67% scored 3
20.	De Visschere et al. (2011)	Nursing homes, Region of Gent in Flanders, Belgium	Complete and partial	Visual	Augsburger and Elahi (1982)	The mean value of plaque index for control group (metal framework) was 2.16 ± 0.91
21.	De Visschere et al. (2012)	Nursing homes, Flanders, Belgium	Complete and partial	Visual	Augsburger and Elahi (1982)	The mean value of plaque index for control group was 2.25 ± 0.94
22.	dos Santos et al. (2010)	Community, Carlos Barbosa, Brazil	Complete dentures	Visual	Ambjørnsen et al. (1982)	The mean value of visual index for control group was 2.47 ± 1.84
23.	Ryu et al. (2010)	Community, Tokyo Dental College, Chiba, Japan	Complete	Visual	Schubert and Schubert index (1978), with minor modifications	The mean value of visual index was 44.37% (expressed as percentage)
24.	Chughtai et al. (2013)	Community, Khyber College of Dentistry, Peshawar	Complete and partial	Visual	Russell et al. (1999)	8% scored 0 (no plaque noted), while 92% scored 2 (ranging from 25% to 50%)
25.	Sloane et al. (2013)	Nursing homes, in North Caroline	Complete and partial	Visual	Augsburger and Elahi (1982)	The mean value of visual index for control group was 2.9 ± 0.9
26.	van der Putten et al. (2013)	Nursing homes, Carehomes in the Netherlands	Complete and partial	Visual	Augsburger and Elahi (1982)	The mean value of visual index for control group was 2.85 ± 0.94
27.	Zenthöfer et al. (2013)	Nursing homes, long‐term carehomes in South‐West Germany	Complete and partial	Visual	Wefer's DHI	The mean value of DHI for control group was 62.4% (expressed as percentage)
28.	Marinoski et al. (2014)	Community, University of Novi Sad, Novi Sad, Serbia	Complete and partial	Visual	Budtz‐Jørgensen and Bertram (1970)	36.7% of control healthy patients scored excellent, while 63.3% scored poor/bad
29.	Almas et al. (2015)	Community, College of Dentistry, King Saud University	Complete	Visual	ASKD‐DPI (Almas, Salameh, Kutkut, and Doubali‐Denture Plaque Index)	30% scored low, 50% scored moderate, and 20% scored high plaque index
30.	Khanagar et al. (2015)	Nursing homes, Bangalore City, India.	N/A	Visual	Augsburger and Elahi (1982)	The mean value of visual index was 3.15 ± 0.47
31.	Zenthofer et al. (2014)	Nursing homes, 13 long‐term carehomes in southwest Germany	Complete and partial	Visual	Wefer's DHI	The mean value of control group was 82.6% using Wefer's DHI (expressed as percentage)
32.	Almeida et al. (2016)	Community, University of São Paulo	Partial	Visual	Tarbet index	18.82% scored 1, 52.69% scored 2, 24.19% scored 3, and 4.3% scored 4.
33.	Zenthöfer et al. (2016)	Nursing home, Southwestern Germany	N/A	Visual	Wefer's DHI	The mean value of control group was 86.1% using Wefer's DHI (expressed as percentage)
34.	Nihtilä et al. (2017)	Nursing homes, Home care clients from Eastern and Central Finland	Complete and partial	Visual	Binary score (good/not good)	63.2% of the control group have good denture hygiene score
35.	Zimmerman et al. (2017)	Nursing homes in North Carolina	Complete and partial	Visual	Augsburger and Elahi (1982)	The mean value of visual index for control group was 2.2 ± 1.2
36.	Weintraub et al. (2018)	Nursing homes in Chapel Hill	N/A	Visual	Augsburger and Elahi (1982)	The mean value of visual index for control group was 2.2 ± 1.3
37.	Srinivasan et al. (2019)	Hospital settings, Geriatric wards in a university hospital setting (Trois‐Chêne Hospital) in Geneva, Switzerland	Complete and partial	Visual	Ambjornsen et al. (1982)	The mean value of visual index was 2 ± 0.9
38.	Klotz et al. (2020)	Nursing homes, Germany	N/A	Visual	Wefer's DHI	The mean value of control group was 54.50% plaque assessment using Wefer's DHI (it is expressed as percentage)
39.	Alqarni et al. (2021)	Hospital setting, North India	Complete dentures	Visual	Jeganathan et al. (1996)	For control group (1 month), 5.3% scored 1, 30.6% scored 2, 36% scored 3, and 28% scored 4, while for control group (24 months), 12% scored 1, 26.67% scored 2, 34.67% scored 3, and 26.67% scored 4
40.	Klotz et al. (2021)	Nursing homes, Heidelberg, Germany	N/A	Visual	Wefer's DHI	The mean value of control group was 54% plaque assessment using Wefer's DHI (expressed as percentage)
41.	Ng et al. (2021)	Community, Faculty of Dentistry, Universiti Kebangsaan Malaysia	Partial	Planimetry: Point counting	Jeganathan et al. (1996)	The mean value of visual index was 3.3 ± 0.8
42.	Able et al. (2022)	Community, ILAPEO College, Brazil	Implant‐ retained dentures	Visual	Mombelli et al. (1987)	5.2% absence, 21.0% visualization with probe only, 57.9% clinically visible, and 15.8% abundant
43.	Valencia‐Heredia et al. (2022)	Community, Dental Center at Universidad de San Martín de Porres, Lima, Perú	Complete and partial	Visual	Vigild's criteria	11.9% scored poor, 68.7% scored fair, and 19.45% scored good
44.	Bleiel et al. (2023)	Nursing homes in Germany	N/A	Visual	Wefer's DHI	Based on D0 main examiner results, the mean value was 9.4 (expressed on a scale from 1 to 10) using Wefer's DHI
45.	Chan et al. (2023)	Community, The University of Hong Kong	Partial	Visual	Mylonas et al. (2014)	The mean value of visual index for group I was 2.77 ± 0.75
46.	Lim, Pan, et al. (2023)	Community, The University of Hong Kong	Complete and partial	Visual	Mylonas et al. (2014)	1% scored 0, 52.65 scored 1, 32% scored 2, 8.2% scored 4, and 6.2% scored 4
47.	Viebranz et al. (2023)	Hospital settings, Geriatric Medicine, Jena University Hospital, Germany	Complete and partial	Visual	Wefer's DHI	10.10% for all surfaces using Wefer's DHI

Abbreviations: DHI, denture hygiene index; N/A, not available.

**Table 2:** Characteristics of included studies (computerized planimetric assessment) in the meta‐analysis.

No.	Study	Location of the study	Sample size	Type of the removable prosthesis	Computer‐assisted planimetric assessment		Mean (%)	Standard deviation (%)
1.	Andrucioli et al. (2004)	Community, University of São Paulo, Brazil	24	Complete	ImageTool	Software ImageTool ‐ Windows, 2.02	58.03	22.67
2.	Panzeri et al. (2009)	Community, Ribeirão Preto Dental School, Brazil	60	Complete	ImageTool	ImageTool 2.02	30.29	21.25
3.	Paranhos et al. (2010)	Community, Ribeirão Preto Dental School, Brazil	62	Complete	ImageTool	ImageTool 2.02	36.70	24.10
4.	Souza et al. (2010)	Community, Ribeirão Preto Dental School, Brazil	53	Complete	ImageTool	ImageTool 2.02	6.88	7.45
5.	Kammers et al. (2015)	Community, Cascavel‐Paraná, Brazil	16	Complete	ImageTool	ImageTool software	55.64	8.53
6.	Abi Nader et al. (2015)	Community, McGill University, Montreal, Quebec, Canada	20	Implant‐ retained dentures	ImageJ	ImageJ 1.45s	28.30	8.40
7.	Al Jaghsi et al. (2018)	Community, University Medicine Greifswald, Germany	34	One or two double‐ crowned retained PRDP	Adobe Photoshop	Adobe Photoshop CS5 Extended 12	48.70	19.67
8.	Arruda et al. (2018)	University of São Paulo	10	Complete	ImageTool	ImageTool 3.0	43.05	7.32
9.	Araujo et al. (2022)	Community, Ribeirão Preto Dental School, Brazil	99	Complete	NIS‐Elements BR	NIS‐Elements BR; Nikon Instruments Inc	22.94	19.27
10.	Chan et al. (2023)	Community, The University of Hong Kong	64	Partial	Adobe Photoshop	Adobe Photoshop	47.25	20.80
11.	Gong et al. (2023)	Community, Department of Oral Implantology, Peking University School and Hospital of Stomatology, China	20	Implant‐ retained dentures	ImageJ	ImageJ 1.52	43.70	15.30
12.	Lim, Pan, et al. (2023)	Community, The University of Hong Kong	97	Complete and partial	Python	Python OpenCV program	24.79	19.78

### 3.1 Assessment of Methodological Quality and Risk of Bias

All included studies were evaluated using the JBI critical appraisal checklist (Appendix 3). The studies rated as “Yes” were a maximum of 100% (12 studies) and a minimum of 55.6% (1 study). The studies were rated as “no” and “unclear” and had a maximum report rate of 33.3% (6 studies) and 11.1% (13 studies), respectively.

### 3.2 Prosthesis Plaque Quantification Methods

I. Planimetric methods:
  The utilization of planimetric plaque assessment techniques for determining plaque surface coverage is increasing, typically represented as a relative plaque area calculation on the prostheses, namely percentage plaque index (PPI) or percentage plaque area coverage (PPC), which indicates the proportion of the surface obscured by visible plaque. In this review, 14 studies using different planimetric assessment methods or software including ImageTool Software (Andrucioli et al. 2004; Kammers et al. 2015; Panzeri et al. 2009; Paranhos et al. 2010; Souza et al. 2010; Arruda et al. 2018), ImageJ Software (Abi Nader et al. 2015; Gong et al. 2023), NIS‐Elements BR; Nikon Instruments Inc. (Araujo et al. 2022), Adobe Photoshop (Al Jaghsi et al. 2018; Chan et al. 2023; Gruender et al. 2021), Python OpenCV Programming Language (Lim, Pan, et al. 2023), and point counting technique (Ng et al. 2021; Ambjørnsen and Rise 1985) were included. Ultimately, 12 studies using computer‐assisted planimetric assessments were included in the meta‐analysis of the prosthesis cleanliness status of adult patients wearing removable dental prostheses.
II. Visual assessment methods:

Visual assessments of removable prosthesis plaque involved staining using a disclosing agent, with erythrosine being the most commonly used agent in these assessments. In this review, 45 studies adopted various visual indices for removable prosthesis plaque measurement.

- The Budtz‐Jörgensen and Bertram index (Kulak‐Ozkan et al. 2002; Marinoski et al. 2014; Budtz‐Jörgensen 1977; Budtz‐Jjörgensen and Kaaber 1986; Budtz‐Jørgensen et al. 1983; Budtz‐Jørgensen and Bertram 1970) was adopted in studies conducted in community hospitals. This index categorizes plaque coverage on the prosthesis fitting surface. The classification system includes (i) excellent (little to no plaque or only a few isolated spots); (ii) fair (<50% of the prosthesis base covered); (iii) poor (≥50% of the prosthesis base covered). A marginally more precise method was documented later in 1978, where the extent of prosthesis plaque on the fitting surface was graded as follows: 0 (not visible); 1+ (covering less than one‐third); 2+ (covering between one‐third and two‐thirds); 3+ (covering more than two‐thirds of the surface). A similar index was later adopted, which simply excluded the score of 3 (Peltola et al. 2007).
- The Augsburger and Elahi index (Khanagar et al. 2015; De Visschere et al. 2011, 2012, 2006; Weintraub et al. 2018; van der Putten et al. 2013; Zimmerman et al. 2017; Sloane et al. 2013; Augsburger and Elahi 1982; Peltola et al. 2004) was applied in long‐term care facilities. The maxillary prosthesis surface was divided into eight sections, comprising four buccal surfaces and four fitting surfaces. The average plaque score is determined by calculating the sum of all eight areas: 0 (absence of plaque); 1 (minimal plaque, covering 1%–25% of the area); 2 (moderate plaque, covering 26%–50% of the area), 3 (substantial plaque, covering 51%–75% of the area); 4 (extensive plaque, covering 76%–100% of the area). The prosthesis plaque index developed by Jeganathan et al. (1996) (Barbeau et al. 2003; Alqarni et al. 2021), which was modified from the Tarbet index (Knezović Zlatarić et al. 2002; Celić et al. 2001; Fraga De Almeida et al. 2016; Tarbet et al. 1984; Badawi et al. 1986a, 1986b), quantifies the plaque area similarly to the Augsburger and Elahi index. However, this method assessed only the maxillary palatal fitting surface. Notably, the fitting surface was sectioned into 4 approximately equal parts by drawing an anteroposterior line at the midline and another line perpendicular to the midline at about the premolar region using the Tarbet index. Each quadrant was scored as described previously, and a total prosthesis plaque score was obtained by summing the quadrants’ scores (maximum score = 16). Similar grading was adopted for different prosthesis surfaces (Keng and Lim 1996). Later, a few studies (Mylonas et al. 2014; Lim, Pan, et al. 2023; Chan et al. 2023) adopted it as a prosthesis cleanliness index by assessing the fitting surface only, or the amount of plaque was measured on a scale of 0 to 3, excluding a score of 4 (Russell et al. 1999; Chughtai et al. 2013).
- For the Ambjornsen index (Al‐Dwairi 2008; dos Santos et al. 2010; Srinivasan et al. 2019; Ambjørnsen et al. 1984, 1982), plaque was documented in five distinct regions, corresponding to the following areas: (i) the incisive papilla; (ii) the rearmost sections of both maxillary tuberosities; (iii) two sections situated 1 cm laterally from the palate's midline at the midpoint between the impression of the superior labial frenum and the most posterior point on the median line of the maxillary prosthesis. Each region was confined to a circle with a diameter of 1 cm. The chosen locations of these five areas were deemed to be representative of the fitting surface. Plaque quantity was assessed using a four‐tiered scoring system: 0 (no plaque); 1 (plaque visible only by scraping with a blunt instrument); 2 (moderate accumulation of visible plaque); 3 (abundance of plaque). Later, this index was adopted for the fitting surface of implant prostheses by grading it similarly (Mombelli et al. 1987; Able et al. 2022).
- McCabe et al. (1996) examined prosthesis plaque coverage using a set of standard prostheses that were painted to simulate plaque scores in the range of 0–10.
- Wefer's Denture Hygiene Index was employed in various studies (Zenthöfer et al. 2014, 2016, 2013; Klotz et al. 2021, 2020; Bleiel et al. 2023; Viebranz et al. 2023; Wefers 1999) in long‐term care facilities and hospitals. Prostheses were stained with a plaque indicator (Plaque Test; Ivoclar Vivadent, Schaan, Liechtenstein), rinsed with water, and examined for plaque‐positive sites using a polymerization light (Bluephase; Ivoclar Vivadent). Subsequently, plaque‐positive areas were counted separately for each prosthesis and divided by 10 (total possible sites), resulting in a score ranging from 0% to 100%.
- Vigild's criteria (Miyazaki et al. 1992; Valencia‐Heredia et al. 2022; Vigild 1987) classified the bacterial plaque on the prosthesis fitting surface as 0 (no visible plaque), 1 (moderate accumulation of visible plaque), and 2 (visible plaque was abundant).
- Nihtilä et al. (2017) employed a binary score of good or not good.
- The ASKD‐DPI (Almas‐, Salameh‐, Kutkut‐, and Doubali‐Denture Plaque Index) (Almas et al. 2015) was used for plaque quantification of complete prosthesis fitting surfaces, which were divided into 10 areas. Each area was scored at 10%, and the total percentage was 100%, reflecting the percentage of the fitting surfaces of maxillary and mandibular complete prostheses containing plaque.
- Muenchinger (1975) evaluated plaque and calculus presence on all teeth and adjacent flanges, as well as on three selected tissue surfaces of each maxillary and mandibular prosthesis, scoring them as either present or absent. This resulted in a possible total score of 63 for either plaque or calculus on each of the maxillary and mandibular prostheses.
- The Schubert and Shubert index (Schubert 1978; Ryu et al. 2010) quantified prosthesis plaque on the fitting surface, which was divided into nine areas. Each area was graded as follows: 0 (no plaque), 1 (a few spots of plaque), 2 (<50% covered by plaque), and 3 (≥50% covered by plaque). The prosthesis hygiene index was calculated based on the sum of individual scores divided by the sum of evaluated areas.

### 3.3 Percentage Plaque Coverage Area

**Figure 2:**
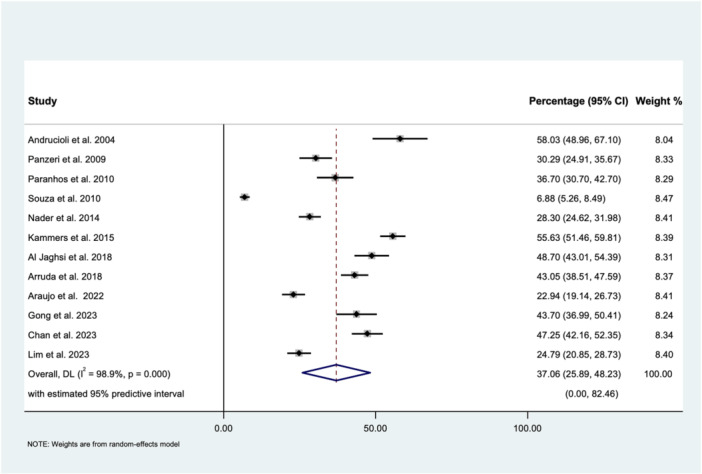
Forest plot of the estimated pooled percentage plaque coverage area, as measured using the computerized planimetric assessments, using the random‐effects (DL, DerSimonian and Laird) model.

**Figure 3:**
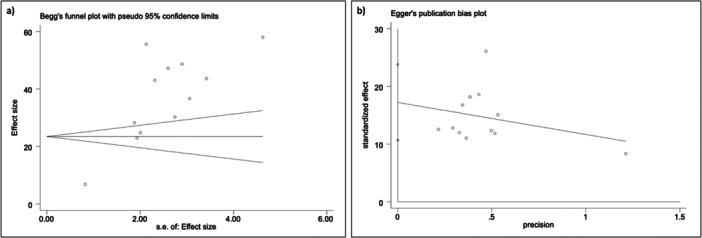
Tests for publication bias (a) Begg's Test and (b) Egger's Test.

**Figure 4:**
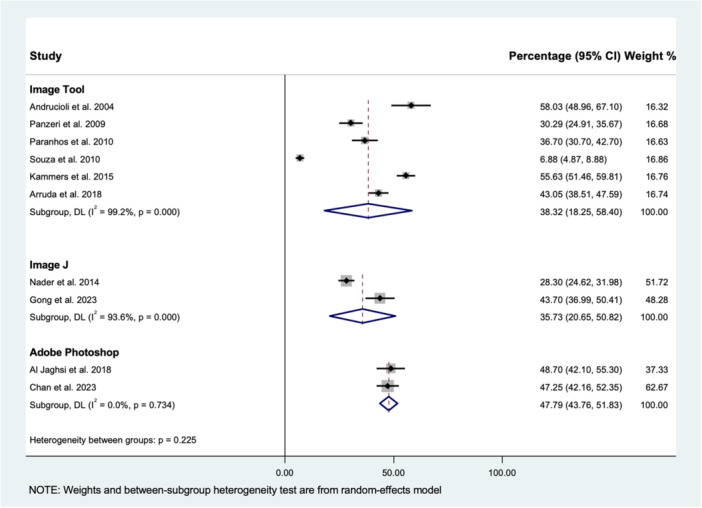
Forest plot of the estimated pooled percentage plaque coverage area, based on computer software using the random‐effects (DL, DerSimonian and Laird) model.

The estimated pooled percentage plaque coverage area, as measured using the computerized planimetric assessments, was 37.06% (95% CI: 25.89%–48.23%) with a considerable heterogeneity (*I* ^2^ = 98.9%, *p* < 0.001) and 95% predictive interval; (0.00, 82.46%) (Figure 2). The Begg (*p* = 0.150) and Egger (*p* = 0.344) tests suggested that there was no publication bias (Figure 3). A sensitivity analysis was performed by excluding studies that assessed prosthesis surfaces other than the fitting surface. The estimated pooled percentage plaque coverage area was 38.85% (95% CI: 31.04%–46.66%), suggesting there was a similar result found between the two analyses (Appendix 4). Subgroup analysis was performed according to the computer software, including ImageTool, ImageJ, and Adobe Photoshop (Figure 4). Their estimated pooled percentages of the plaque coverage area using ImageTool, ImageJ, and Adobe Photoshop were 38.3% (95% CI: 18.25%–58.40%), 35.73% (95% CI: 20.65%–50.82%), and 47.79% (95% CI: 43.76%–51.83%), respectively. The heterogeneity for the Adobe Photoshop group was reduced to “might not be important” (*I* ^2^ = 0%, *p* = 0.734).

## 4 Discussion

In the past, systematic reviews have primarily focused on evaluating the effectiveness of removable prosthesis cleaning interventions. However, understanding various prosthesis plaque quantification methods and the overall state of prosthesis cleanliness in the community is essential for promoting public health and safety, and informing the currently available prosthesis plaque area coverage assessment methods (visual and planimetric). Therefore, this systematic review aimed to summarize the existing evidence on removable prosthesis plaque area coverage assessment methods, as well as conduct a meta‐analysis of the cleanliness status of adult patients wearing removable dental prostheses, as quantified using computer‐assisted planimetric assessments.

While computerized planimetric assessments are now common, the majority of evidence on prosthesis plaque assessments has primarily focused on visual index assessments rather than planimetric assessments. Published evidence on planimetric assessments is limited, with 14 studies included in the current review. In addition, various software or methods were employed, including ImageTool Software (Andrucioli et al. 2004; Kammers et al. 2015; Panzeri et al. 2009; Paranhos et al. 2010; Souza et al. 2010; Arruda et al. 2018), ImageJ Software (Abi Nader et al. 2015; Gong et al. 2023), NIS‐Elements BR; Nikon Instruments Inc. (Araujo et al. 2022), Adobe Photoshop (Al Jaghsi et al. 2018; Chan et al. 2023; Gruender et al. 2021), Python OpenCV Programming Language (Lim, Pan, et al. 2023), and point counting technique (Ng et al. 2021; Ambjørnsen and Rise 1985). As a result, computer‐assisted planimetric assessments are recommended to improve analysis precision by allowing linear measurements, improved reproducibility, increased objectivity, and better discriminatory capabilities, which are critical for research studies. Additionally, this technique has been reported to be more reliable and objective than visual plaque indices (Lim, Pan, et al. 2023). The fundamental concept involves capturing a prosthesis image and employing computational software to quantify the surface area in pixels. Over time, this approach has evolved to become more automated and less dependent on subjective interpretation. Conventional image‐editing software primarily employed the RGB (red, green, blue) color model for image analysis, which dictated the combinations and intensities needed to generate specific hues. In contrast, studies reported that HSV (hue, saturation, and value) color space was an alternative approach with high accuracy for measuring plaque area coverage (Lim, Pan, et al. 2023; Carter et al. 2004). Effective planimetric assessments necessitate high‐resolution images with precise focus, devoid of flash anomalies, consistent image dimensions, and distinct separation of image elements that can be discerned by the software. Digital images can be stored and analyzed at a later time, facilitating longitudinal comparisons, reproducibility assessments, and double‐blind investigations. Notably, the development of more automated systems for image acquisition using mobile phones and plaque quantification using planimetric assessments will be beneficial for routine clinical examination (Lim, Pan, et al. 2023).

Various visual plaque indices were commonly adopted in clinical studies for prosthesis plaque area measurements. A total of 43 studies employed visual plaque indices and were included in the present review. Visual indices varied in terms of prosthesis surface assessment, ordinal scale, application of plaque disclosing agent, and area division within a surface, allowing for a more comprehensive analysis of plaque distribution on removable prostheses. Therefore, none of the visual indices has gained widespread acceptance among researchers and dentists. The biggest advantage of visual plaque indices in clinical studies is that prosthesis plaque area coverage can be measured simply and quickly, which can be beneficial for epidemiological or audit studies (Mylonas et al. 2014). However, drawbacks include subjective evaluation, reduced reproducibility across studies, and lower accuracy and sensitivity. Prosthesis cleanliness for patients is rarely monitored and assessed as a routine examination of their oral hygiene status, not as common as plaque and bleeding scores. Possibly, a standardized visual plaque index should be adopted, as prosthesis hygiene is often overlooked by dental practitioners (Ng et al. 2022). In this review, one of the most commonly employed ordinal scores was as follows: score 0 (absence of plaque), 1 (minimal plaque, covering 1%–25% of the area), 2 (moderate plaque, covering 26%–50% of the area), 3 (substantial plaque, covering 51%–75% of the area), and 4 (extensive plaque, covering 76%–100% of the area). However, the main difference among studies was the surfaces selected for plaque area coverage assessment.

Due to the accuracy of computer‐assisted planimetric assessments, removable prosthesis cleanliness was further analyzed to investigate the general hygiene status of adult patients wearing removable dental prostheses in the community. The overall estimated pooled PPC, as measured by computerized planimetric assessments, was 37.06%. These moderately stained prostheses can be considered unclean, although some studies have reported even higher scores (Mylonas et al. 2014; Lim, Lee, et al. 2025). To align with commonly used visual scoring systems, a PPC of 37% corresponds to a score of 2 on the Prosthesis Cleanliness Index (Mylonas et al. 2014), 2 out of 4 on the Augsburger and Elahi Index (Augsburger and Elahi 1982), and is graded as “fair” on the Budtz‐Jörgensen and Bertram Index (Budtz‐Jørgensen and Bertram 1970). Notably, subgroup analysis for other image editing software demonstrated a similar result, except Adobe Photoshop recorded nearly 50% prosthesis plaque coverage with low heterogeneity. Therefore, prosthesis cleanliness among adult patients wearing removable dental prostheses was considered poor and should be monitored regularly. Poor prosthesis hygiene caused by heavy biofilm deposition has been suggested to be associated with various oral and systemic health problems, including denture stomatitis, caries, periodontal diseases, halitosis, bacterial endocarditis, gastrointestinal infections, and respiratory diseases (Coulthwaite and Verran 2007; Lim, Li, et al. 2025; Lim et al. 2024b; Wong et al. 2024). As reported by multiple reviews, consensus, and clinical trials (de Souza et al. 2009; Lim, Lee, et al. 2025; Felton et al. 2011; Lim, Huang, et al. 2025; Lim, Burrow, et al. 2024), a combination of mechanical (manual toothbrush or ultrasonic cleaning) and chemical approaches remains the most recommended intervention for prosthesis cleaning.

A high degree of heterogeneity (*I*² = 98.9%) was observed among the included studies for the overall estimated pooled PPC. Subgroup meta‐analysis showed that studies using Adobe Photoshop had much lower heterogeneity compared with studies using other image editing software. The low heterogeneity within the Adobe Photoshop group suggests that variations in software used may be a major source of overall heterogeneity. Other possible sources of heterogeneity include study design, type of prosthesis, prosthesis surface assessed, image acquisition protocols, study population, and study setting. Prediction intervals were also used to estimate the range of true treatment effects, which helps to better interpret the clinical implications of the observed heterogeneity and to avoid misinterpretation of meta‐analysis results (Lim, Tan, et al. 2023). The prediction intervals presented in this study provide a more clinically meaningful estimate for future prosthesis hygiene status than the usual summary effect. For instance, while the observed heterogeneity of computerized planimetric assessment was considerable, the pooled percentage plaque coverage area could be predicted to be as low as 0% and as high as 82%.

This systematic review adopted a JBI critical appraisal checklist for quality and risk of bias assessment, reporting that the quality level of included studies was moderate to good. Notably, there was no statistically significant difference in publication bias as assessed using Begg's and Egger's tests, suggesting that the present review included a balanced representation of studies, regardless of their outcomes or effect sizes. In addition, the sensitivity analysis showed that the result is robust. Some practical recommendations can be made regarding plaque assessment for removable prostheses. Computerized planimetric assessment using digital single‐lens reflex images remains the gold standard; however, its use is primarily limited to research settings. To facilitate wider clinical adoption of plaque assessment for removable prostheses, more automated methods, such as mobile phone photography combined with artificial intelligence, should be developed. In addition, this review found that PPC measured on the fitting surface was more standardized and comparable across studies than the various visual indices reported in the literature. Therefore, PPC on the fitting surface should be considered as an outcome measure in future clinical studies.

## 5 Conclusions

The outcomes of this review revealed poor removable prosthesis cleanliness among adult patients, which may potentially be related to an increased risk of oral and systemic infections in individuals wearing removable prostheses. There were no standard removable prosthesis plaque quantification methods for research and clinical use. Most studies adopted traditional visual indices for removable prosthesis plaque assessment, suggesting that a more automated computerized planimetric assessment should be considered to improve analysis precision.

## Author Contributions

**Tong Wah Lim:** conceptualization, data curation, formal analysis, investigation, visualization, writing – original draft, writing – review and editing. **Maxstein M. Abuzaid:** data curation, formal analysis, investigation, writing – original draft. **Jade Yuen Kei Wong:** data curation, formal analysis, investigation. **Kar Yan Li:** data curation, formal analysis, visualization. **Michael Francis Burrow:** conceptualization, formal analysis, writing – review and editing. **Colman McGrath:** conceptualization, formal analysis, writing – review and editing.

## Ethics Statement

The authors have nothing to report.

## Conflicts of Interest

The authors declare no conflicts of interest.

## Supporting information


**Appendix 1:** Search strategy. **Appendix 2:** Joanna Briggs Institute (JBI) critical appraisal checklist questions. **Appendix 3:** Quality assessment of included studies, Joanna Briggs Institute critical appraisal checklist. **Appendix 4:** Sensitivity analysis after excluding studies that assessed denture surfaces other than the fitting surface.

## Data Availability

The data that support the findings of this study are available from the corresponding author upon reasonable request.
