# Supplementary material for: Quantification of Removable Prosthesis Plaque Area Coverage Among Adult Patients: A Systematic Review and Meta‐Analysis
Source: Clin Exp Dent Res. 2026 Feb 25;12(2):e70319. doi: 10.1002/cre2.70319 (PMC12935217; doi:10.1002/cre2.70319)

**Supplementary File**

**Quantification of removable prosthesis plaque area coverage among adult patients: A systematic review and meta-analysis**

Tong Wah Lim, Maxstein M. Abuzaid, Jade Yuen Kei Wong, Kar Yan Li, Michael Francis Burrow, Colman McGrath

Appendix 1 Search strategy

Appendix 2 Joanna Briggs Institute (JBI) critical appraisal checklist questions.

Appendix 3 Quality assessment of included studies, Joanna Briggs Institute critical appraisal checklist.

Appendix 4 Sensitivity analysis after excluding studies that assessed denture surfaces other than fitting surface.

Appendix 1. Search strategy.

PubMed:

Searched on 30 Oct 2023

Results: 1,984

| # | Search History in PubMed | Results |
| --- | --- | --- |
| #1 | "Dental Plaque Index"[Mesh:NoExp] | 5,643 |
| #2 | “dental plaque index*”[Title/Abstract] OR “dental plaque indice*”[Title/Abstract] OR (dental[Title/Abstract] AND plaque[Title/Abstract] AND (index*[Title/Abstract] OR indice*[Title/Abstract])) OR “plaque coverage” [Title/Abstract] OR (plaque[Title/Abstract] AND coverage[Title/Abstract]) OR “biofilm coverage”[Title/Abstract] OR (biofilm[Title/Abstract] AND coverage[Title/Abstract]) OR “plaque accumulation”[Title/Abstract] OR (plaque[Title/Abstract] AND accumulation[Title/Abstract]) OR “biofilm accumulation”[Title/Abstract] OR (biofilm[Title/Abstract] AND accumulation[Title/Abstract]) OR “plaque scor*”[Title/Abstract] OR (plaque[Title/Abstract] AND scor*[Title/Abstract]) OR “biofilm scor*”[Title/Abstract] OR (biofilm[Title/Abstract] AND scor*[Title/Abstract]) | 22,096 |
| #3 | ((dry[Title/Abstract] OR wet[Title/Abstract]) AND “weight measure*”[Title/Abstract]) OR “biochemical assay*”[Title/Abstract] OR “oxygen consumption assay*”[Title/Abstract] OR “microbiological count*”[Title/Abstract] OR “visual indice*”[Title/Abstract] OR “planimetric assessment*”[Title/Abstract] OR “digital image analysis”[Title/Abstract] OR (visual[Title/Abstract] AND plaque[Title/Abstract] AND scor*[Title/Abstract]) OR (computerized[Title/Abstract] AND image[Title/Abstract] AND assessment[Title/Abstract]) OR “image processing software”[Title/Abstract] OR “software image tool”[Title/Abstract] OR (analogue[Title/Abstract] AND plaque[Title/Abstract] AND score[Title/Abstract]) OR “additive Index”[Title/Abstract] OR (Silness[Title/Abstract] AND Löe[Title/Abstract] AND plaque[Title/Abstract] AND index[Title/Abstract]) OR (Turesky[Title/Abstract] AND index[Title/Abstract]) OR (Quigley[Title/Abstract] AND Hein[Title/Abstract] AND plaque[Title/Abstract] AND index[Title/Abstract]) OR (Bonded[Title/Abstract] AND bracket[Title/Abstract] AND plaque[Title/Abstract] AND index[Title/Abstract]) OR “Lobene stain index”[Title/Abstract] OR “Navy Plaque Index”[Title/Abstract] OR (Rustogi[Title/Abstract] AND Index[Title/Abstract]) OR “Quantitative Light-induced Fluorescence”[Title/Abstract] | 16,345 |
| #4 | #1 OR #2 OR #3 | 40,157 |
| #5 | ("Dentures"[Mesh]) OR "Dental Prosthesis"[Mesh] | 116,481 |
| #6 | denture*[Title/Abstract] OR "false teeth"[Title/Abstract] OR “false tooth”[Title/Abstract] OR "artificial teeth"[Title/Abstract] OR “artificial tooth”[Title/Abstract] OR "dental prosthe*"[Title/Abstract] | 33,243 |
| #7 | #5 OR #6 | 124,431 |
| #8 | #4 AND #7 | 2,196 |
| #9 | #4 AND #7 Filters: Humans | 1,984 |

(("Dental Plaque Index"[MeSH Terms:noexp] OR ("dental plaque index*"[Title/Abstract] OR "dental plaque indice*"[Title/Abstract] OR ("dental"[Title/Abstract] AND "plaque"[Title/Abstract] AND ("index*"[Title/Abstract] OR "indice*"[Title/Abstract])) OR "plaque coverage"[Title/Abstract] OR ("plaque"[Title/Abstract] AND "coverage"[Title/Abstract]) OR "biofilm coverage"[Title/Abstract] OR ("biofilm"[Title/Abstract] AND "coverage"[Title/Abstract]) OR "plaque accumulation"[Title/Abstract] OR ("plaque"[Title/Abstract] AND "accumulation"[Title/Abstract]) OR "biofilm accumulation"[Title/Abstract] OR ("biofilm"[Title/Abstract] AND "accumulation"[Title/Abstract]) OR "plaque scor*"[Title/Abstract] OR ("plaque"[Title/Abstract] AND "scor*"[Title/Abstract]) OR "biofilm scor*"[Title/Abstract] OR ("biofilm"[Title/Abstract] AND "scor*"[Title/Abstract])) OR ((("dry"[Title/Abstract] OR "wet"[Title/Abstract]) AND "weight measure*"[Title/Abstract]) OR "biochemical assay*"[Title/Abstract] OR "oxygen consumption assay*"[Title/Abstract] OR "microbiological count*"[Title/Abstract] OR "visual indice*"[Title/Abstract] OR "planimetric assessment*"[Title/Abstract] OR "digital image analysis"[Title/Abstract] OR ("visual"[Title/Abstract] AND "plaque"[Title/Abstract] AND "scor*"[Title/Abstract]) OR ("computerized"[Title/Abstract] AND "image"[Title/Abstract] AND "assessment"[Title/Abstract]) OR "image processing software"[Title/Abstract] OR "software image tool"[Title/Abstract] OR ("analogue"[Title/Abstract] AND "plaque"[Title/Abstract] AND "score"[Title/Abstract]) OR "additive Index"[Title/Abstract] OR ("Silness"[Title/Abstract] AND "Loe"[Title/Abstract] AND "plaque"[Title/Abstract] AND "Index"[Title/Abstract]) OR ("Turesky"[Title/Abstract] AND "Index"[Title/Abstract]) OR ("Quigley"[Title/Abstract] AND "Hein"[Title/Abstract] AND "plaque"[Title/Abstract] AND "Index"[Title/Abstract]) OR ("Bonded"[Title/Abstract] AND "bracket"[Title/Abstract] AND "plaque"[Title/Abstract] AND "Index"[Title/Abstract]) OR "Lobene stain index"[Title/Abstract] OR "Navy Plaque Index"[Title/Abstract] OR ("Rustogi"[Title/Abstract] AND "Index"[Title/Abstract]) OR "Quantitative Light-induced Fluorescence"[Title/Abstract])) AND ("Dentures"[MeSH Terms] OR "Dental Prosthesis"[MeSH Terms] OR ("denture*"[Title/Abstract] OR "false teeth"[Title/Abstract] OR "false tooth"[Title/Abstract] OR "artificial teeth"[Title/Abstract] OR "artificial tooth"[Title/Abstract] OR "dental prosthe*"[Title/Abstract]))) AND (humans[Filter])

Ovid Medline

Ovid MEDLINE(R) and Epub Ahead of Print, In-Process, In-Data-Review & Other Non-Indexed Citations, Daily and Versions 1946 to October 27, 2023

Searched on 30 October 2023

Results: 1556

exp animals/ not humans.sh.

| # | Search History in Ovid Medline | Results |
| --- | --- | --- |
| 1 | Dental Plaque Index/ | 5643 |
| 2 | ("dental plaque index*" or " dental plaque indice*" or (dental adj6 plaque adj6 (index* or indice*)) or "plaque coverage" or (plaque adj6 coverage) or "biofilm coverage" or (biofilm adj6 coverage) or "plaque accumulation" or (plaque adj6 accumulation) or "biofilm accumulation" or (biofilm adj6 accumulation) or "plaque scor*" or (plaque adj6 scor*) or "biofilm scor*" or (biofilm adj6 scor*)).ab,ti. | 8071 |
| 3 | (((dry or wet) adj6 "weight measure*") or "biochemical assay*" or "oxygen consumption assay*" or "microbiological count*" or "visual indice*" or "planimetric assessment*" or "digital image analysis" or (visual adj6 plaque adj6 scor*) or (computerized adj6 image adj6 assessment) or "image processing software" or "software image tool" or (analogue adj6 plaque adj6 score) or "additive Index" or (Silness adj6 L*e adj6 plaque adj6 index) or (Turesky adj6 index) or (Quigley adj6 Hein adj6 plaque adj6 index) or (Bonded adj6 bracket adj6 plaque adj6 index) or "Lobene stain index" or "Navy Plaque Index" or (Rustogi adj6 Index) or "Quantitative Light-induced Fluorescence").ab,ti. | 15130 |
| 4 | 1 or 2 or 3 | 26629 |
| 5 | exp Dentures/ or exp Dental Prosthesis/ | 116492 |
| 6 | (denture* or "false teeth" or "false tooth" or "artificial teeth" or "artificial tooth" or "dental prosthe*").ab,ti. | 31262 |
| 7 | 5 or 6 | 124094 |
| 8 | 4 and 7 | 1712 |
| 9 | limit 8 to humans | 1556 |

<https://eproxy.lib.hku.hk/login?url=http://ovidsp.ovid.com/ovidweb.cgi?T=JS&NEWS=N&PAGE=main&SHAREDSEARCHID=7XbabEatnkVULK3QSaaWL7hXugQzv9U12jGcxEc32HzN0VOMbOhNiCtwD84OK5S36>

EMBASE

Embase Classic+Embase 1947 to 2023 October 27

Searched on 30 October 2023

Results: 1333

| # | Search History in Embase | Results |
| --- | --- | --- |
| 1 | plaque index/ | 3828 |
| 2 | ("dental plaque index*" or " dental plaque indice*" or (dental adj6 plaque adj6 (index* or indice*)) or "plaque coverage" or (plaque adj6 coverage) or "biofilm coverage" or (biofilm adj6 coverage) or "plaque accumulation" or (plaque adj6 accumulation) or "biofilm accumulation" or (biofilm adj6 accumulation) or "plaque scor*" or (plaque adj6 scor*) or "biofilm scor*" or (biofilm adj6 scor*)).ab,ti. | 9932 |
| 3 | (((dry or wet) adj6 "weight measure*") or "biochemical assay*" or "oxygen consumption assay*" or "microbiological count*" or "visual indice*" or "planimetric assessment*" or "digital image analysis" or (visual adj6 plaque adj6 scor*) or (computerized adj6 image adj6 assessment) or "image processing software" or "software image tool" or (analogue adj6 plaque adj6 score) or "additive Index" or (Silness adj6 L*e adj6 plaque adj6 index) or (Turesky adj6 index) or (Quigley adj6 Hein adj6 plaque adj6 index) or (Bonded adj6 bracket adj6 plaque adj6 index) or "Lobene stain index" or "Navy Plaque Index" or (Rustogi adj6 Index) or "Quantitative Light-induced Fluorescence").ab,ti. | 19700 |
| 4 | 1 or 2 or 3 | 32281 |
| 5 | exp denture/ or exp tooth prosthesis / | 106361 |
| 6 | (denture* or "false teeth" or "false tooth" or "artificial teeth" or "artificial tooth" or "dental prosthe*").ab,ti. | 31943 |
| 7 | 5 or 6 | 111227 |
| 8 | 4 and 7 | 1450 |
| 9 | limit 8 to humans | 1333 |

https://eproxy.lib.hku.hk/login?url=http://ovidsp.ovid.com/ovidweb.cgi?T=JS&NEWS=N&PAGE=main&SHAREDSEARCHID=3Lm0TvxPUxEmqCVr7jCOSZa8vntdItoSIIyESXYXhuqalHSgCvnlxxqBQNKHHFvwc

Scopus

Searched on 30 October 2023

Results:  1048

| # | Search History in Scopus | Results |
| --- | --- | --- |
| 1 | INDEXTERMS ( "dental plaque index" ) | 5309 |
| 2 | TITLE-ABS (   "dental plaque index*"   ) OR TITLE-ABS (   "dental plaque indice*"   ) OR TITLE-ABS (  dental W/6 plaque W/6 ( index* OR indice* ) ) OR TITLE-ABS (   "plaque coverage" ) OR TITLE-ABS (  plaque W/6 coverage ) OR TITLE-ABS (   "biofilm coverage" ) OR TITLE-ABS (  biofilm W/6 coverage ) OR TITLE-ABS (   "plaque accumulation" ) OR TITLE-ABS (  plaque W/6 accumulation ) OR TITLE-ABS (   "biofilm accumulation" ) OR TITLE-ABS (  biofilm W/6 accumulation ) OR TITLE-ABS (   "plaque scor*" ) OR TITLE-ABS (  plaque W/6 scor* ) OR TITLE-ABS (   "biofilm scor*" ) OR TITLE-ABS (  biofilm W/6 scor* ) | 12580 |
| 3 | TITLE-ABS ( ( dry OR wet ) W/6 "weight measure*" ) OR TITLE-ABS (   "biochemical assay* " ) OR TITLE-ABS (   "oxygen consumption assay*" ) OR TITLE-ABS (   "microbiological count*" ) OR TITLE-ABS ( "visual indice*" ) OR TITLE-ABS (   "planimetric assessment*" ) OR TITLE-ABS (   "digital image analysis" ) OR TITLE-ABS (  visual W/6 plaque W/6 scor* ) OR TITLE-ABS (  computerized W/6 image W/6 assessment ) OR TITLE-ABS (   "image processing software" ) OR TITLE-ABS (   "software image tool" ) OR TITLE-ABS (  analogue W/6 plaque W/6 score ) OR TITLE-ABS (   "additive Index" ) OR TITLE-ABS ( silness W/6 l*e W/6 plaque W/6 index ) OR TITLE-ABS ( turesky W/6 index ) OR TITLE-ABS (  quigley W/6 hein W/6 plaque W/6 index ) OR TITLE-ABS ( bonded W/6 bracket W/6 plaque W/6 index ) OR TITLE-ABS ( "Lobene stain index" ) OR TITLE-ABS ( "Navy Plaque Index" ) OR TITLE-ABS ( rustogi W/6 index ) OR TITLE-ABS ( "Quantitative Light-induced Fluorescence" ) | 23695 |
| 4 | #1 OR #2 OR #3 | 39,139 |
| 5 | INDEXTERMS ( dentures ) OR INDEXTERMS ( "dental prosthesis" ) | 74832 |
| 6 | TITLE-ABS (   "denture*" ) OR TITLE-ABS ( "false teeth" ) OR TITLE-ABS ( "false tooth" ) OR TITLE-ABS ( "artificial teeth" ) OR TITLE-ABS ( "artificial tooth" ) OR TITLE-ABS ( "dental prosthe*" ) | 37292 |
| 7 | #5 OR #6 | 87315 |
| 8 | #4 AND #7 | 1095 |
| 9 | INDEXTERMS ( animals OR animal ) |  |
| 10 | #8 AND NOT #9 | 1048 |

( INDEXTERMS ( "dental plaque index" ) OR TITLE-ABS ( "dental plaque index*" ) OR TITLE-ABS ( "dental plaque indice*" ) OR TITLE-ABS ( dental W/6 plaque W/6 ( index* OR indice* ) ) OR TITLE-ABS ( "plaque coverage" ) OR TITLE-ABS ( plaque W/6 coverage ) OR TITLE-ABS ( "biofilm coverage" ) OR TITLE-ABS ( biofilm W/6 coverage ) OR TITLE-ABS ( "plaque accumulation" ) OR TITLE-ABS ( plaque W/6 accumulation ) OR TITLE-ABS ( "biofilm accumulation" ) OR TITLE-ABS ( biofilm W/6 accumulation ) OR TITLE-ABS ( "plaque scor*" ) OR TITLE-ABS ( plaque W/6 scor* ) OR TITLE-ABS ( "biofilm scor*" ) OR TITLE-ABS ( biofilm W/6 scor* ) OR TITLE-ABS ( ( dry OR wet ) W/6 "weight measure*" ) OR TITLE-ABS ( "biochemical assay* " ) OR TITLE-ABS ( "oxygen consumption assay*" ) OR TITLE-ABS ( "microbiological count*" ) OR TITLE-ABS ( "visual indice*" ) OR TITLE-ABS ( "planimetric assessment*" ) OR TITLE-ABS ( "digital image analysis" ) OR TITLE-ABS ( visual W/6 plaque W/6 scor* ) OR TITLE-ABS ( computerized W/6 image W/6 assessment ) OR TITLE-ABS ( "image processing software" ) OR TITLE-ABS ( "software image tool" ) OR TITLE-ABS ( analogue W/6 plaque W/6 score ) OR TITLE-ABS ( "additive Index" ) OR TITLE-ABS ( silness W/6 l*e W/6 plaque W/6 index ) OR TITLE-ABS ( turesky W/6 index ) OR TITLE-ABS ( quigley W/6 hein W/6 plaque W/6 index ) OR TITLE-ABS ( bonded W/6 bracket W/6 plaque W/6 index ) OR TITLE-ABS ( "Lobene stain index" ) OR TITLE-ABS ( "Navy Plaque Index" ) OR TITLE-ABS ( rustogi W/6 index ) OR TITLE-ABS ( "Quantitative Light-induced Fluorescence" ) ) AND ( INDEXTERMS ( dentures ) OR INDEXTERMS ( "dental prosthesis" ) OR TITLE-ABS ( "denture*" ) OR TITLE-ABS ( "false teeth" ) OR TITLE-ABS ( "false tooth" ) OR TITLE-ABS ( "artificial teeth" ) OR TITLE-ABS ( "artificial tooth" ) OR TITLE-ABS ( "dental prosthe*" ) ) AND NOT ( INDEXTERMS ( animals OR animal ) )

Cochrane Central Register of Controlled Trials (CENTRAL)

The Cochrane Central Register of Controlled Trials (CENTRAL) is a highly concentrated source of reports of randomized and quasi-randomized controlled trials. Most CENTRAL records are taken from bibliographic databases (mainly PubMed and Embase.com), but records are also derived from other published and unpublished sources, including CINAHL, ClinicalTrials.gov and the WHO's International Clinical Trials Registry Platform. CENTRAL first began publication in 1996, but its composite nature means that it does not have an inception (start) date, in the way that other traditional biomedical databases do

Warning: wildcards (* or ?) in quoted phrases will not find all results. Use the NEXT command and nesting to search a phrase with wildcards. Instead of "heart transplant*", use (heart NEXT transplant*).

Searched on 30 October 2023

Results: 421

| # | Search History in CENTRAL | Results |
| --- | --- | --- |
| #1 | MeSH descriptor: [Dental Plaque Index] this term only | 2148 |
| #2 | ("dental plaque index*" or " dental plaque indice*" or (dental near/6 plaque near/6 (index* or indice*)) or "plaque coverage" or (plaque near/6 coverage) or "biofilm coverage" or (biofilm near/6 coverage) or "plaque accumulation" or (plaque near/6 accumulation) or "biofilm accumulation" or (biofilm near/6 accumulation) or "plaque scor*" or (plaque near/6 scor*) or "biofilm scor*" or (biofilm near/6 scor*)):ab,ti | 3183 |
| #3 | (((dry or wet) near/6 "weight measure*") or "biochemical assay*" or "oxygen consumption assay*" or "microbiological count*" or "visual indice*" or "planimetric assessment*" or "digital image analysis" or (visual near/6 plaque near/6 scor*) or (computerized near/6 image near/6 assessment) or "image processing software" or "software image tool" or (analogue near/6 plaque near/6 score) or "additive Index" or (Silness near/6 L*e near/6 plaque near/6 index) or (Turesky near/6 index) or (Quigley near/6 Hein near/6 plaque near/6 index) or (Bonded near/6 bracket near/6 plaque near/6 index) or "Lobene stain index" or "Navy Plaque Index" or (Rustogi near/6 Index) or "Quantitative Light-induced Fluorescence"):ab,ti | 1412 |
| #4 | #1 OR #2 OR #3 | 5351 |
| #5 | MeSH descriptor: [Dentures] explode all trees | 1229 |
| #6 | MeSH descriptor: [Dental Prosthesis] explode all trees | 5629 |
| #7 | (denture* or "false teeth" or "false tooth" or "artificial teeth" or "artificial tooth" or "dental prosthe*"):ab,ti | 2401 |
| #8 | #5 OR #6 OR #7 | 7148 |
| #9 | #4 AND #8 | 425 |
| #10 | [mh “Animals”] NOT [mh “Humans”] | 2966 |
| #11 | #9 NOT #10 (Limit to Trials) | 421 |

Total number of references from five databases: 6342

Duplicate records removed: 3331

Number of records before screening: 3011

Appendix 2. Joanna Briggs Institute (JBI) critical appraisal checklist questions.

| No. | JBI Critical Appraisal Checklist Questions |
| --- | --- |
| Q1 | Was the sample frame appropriate to address the target population? |
| Q2 | Were study participants sampled in an appropriate way? |
| Q3 | Was the sample size adequate? |
| Q4 | Were the study subjects and the setting described in detail? |
| Q5 | Was the data analysis conducted with sufficient coverage of the identified sample? |
| Q6 | Were valid methods used for the identification of the condition? |
| Q7 | Was the condition measured in a standard, reliable way for all participants? |
| Q8 | Was there appropriate statistical analysis? |
| Q9 | Was the response rate adequate, and if not, was the low response rate managed appropriately? |

Appendix 3. Quality assessment of included studies, Joanna Briggs Institute critical appraisal checklist.

| No. | Study | Q1 | Q2 | Q3 | Q4 | Q5 | Q6 | Q7 | Q8 | Q9 | Overall appraisal |
| --- | --- | --- | --- | --- | --- | --- | --- | --- | --- | --- | --- |
| 1. | Muenchinger et al. 1975 | / | / | x | x | / | / | / | / | / | Include |
| 2. | Budtz-Jörgensen et al. 1977 | x | / | x | / | / | / | / | / | / | Include |
| 3. | Ambjørnsen et al. 1984 | x | / | x | / | / | / | / | / | / | Include |
| 4. | Ambjørnsen et al. 1985 | / | / | / | x | / | / | / | / | / | Include |
| 5. | Badawi et al 1986 (a) | x | / | x | / | / | / | / | / | - | Include |
| 6. | Badawi et al 1986 (b) | x | / | x | / | / | / | / | / | - | Include |
| 7. | Budtz-Jörgensen et al. 1986 | / | / | x | x | / | / | / | / | - | Include |
| 8. | Miyazaki et al. 1992 | x | / | x | / | / | / | / | / | / | Include |
| 9. | Jeganathan et al. 1996 | x | / | x | / | / | / | / | / | / | Include |
| 10. | Keng et al. 1996 | / | / | x | / | / | / | / | / | / | Include |
| 11. | McCabe et al. 1996 | x | - | / | x | / | / | / | / | / | Include |
| 12. | Russell et al. 1999 | x | / | x | x | / | / | / | / | / | Include |
| 13. | Celic et al. 2001 | x | / | x | / | / | / | / | / | / | Include |
| 14. | Kulak-Ozkan et al. 2002 | x | / | x | x | / | / | / | / | / | Include |
| 15. | Zlatarić et al. 2002 | x | / | / | / | / | / | / | / | / | Include |
| 16. | Barbeau et al. 2003 | x | / | / | x | / | / | / | / | / | Include |
| 17. | Andrucioli et al. 2004 | x | / | x | / | / | / | / | / | / | Include |
| 18. | De Visschere et al. 2006 | x | / | x | / | / | / | / | / | / | Include |
| 19. | Peltola et al. 2007 | x | / | x | x | / | / | / | / | / | Include |
| 20. | Al-Dwairi et al. 2008 | x | / | x | / | / | / | / | / | / | Include |
| 21. | Panzeri et al. 2009 | / | / | / | / | / | / | / | / | / | Include |
| 22. | De Visschere et al. 2010 (a) | / | / | / | / | / | / | / | / | / | Include |
| 23. | De Visschere et al. 2010 (b) | / | / | / | / | / | / | / | / | / | Include |
| 24. | Dos Santos et al. 2010 | / | / | / | / | / | / | / | / | / | Include |
| 25. | Paranhos et al. 2010 | / | / | / | x | / | / | / | / | / | Include |
| 26. | Ryu et al. 2010 | x | / | x | x | / | / | / | / | / | Include |
| 27. | Souza et al. 2010 | x | / | / | / | / | / | / | / | - | Include |
| 28. | Chughtai et al. 2013 | x | / | x | x | / | / | / | / | / | Include |
| 29. | Sloane et al. 2013 | x | / | x | / | / | / | / | / | - | Include |
| 30. | Van der Putten et al. 2013 | / | / | / | / | / | / | / | / | / | Include |
| 31. | Zenthofer et al. 2013 | / | / | / | x | / | / | / | / | / | Include |
| 32. | Marinoski et al. 2014 | x | / | / | x | / | / | / | / | / | Include |
| 33. | Almas et al. 2015 | x | / | x | / | / | / | / | / | / | Include |
| 34. | Kammers et al. 2015 | x | / | x | / | / | / | - | / | / | Include |
| 35. | Khanagar et al. 2015 | / | / | / | / | / | / | / | / | - | Include |
| 36. | Nader et al. 2015 | x | / | x | / | / | / | / | / | / | Include |
| 37. | Zenthöfer et al. 2015 | x | / | / | x | / | / | / | / | - | Include |
| 38. | Fraga De Almeida et al. 2016 | x | / | x | / | / | / | / | / | / | Include |
| 39. | Zenthöfer et al. 2016 | x | / | / | x | / | / | / | / | - | Include |
| 40. | Nihtila et al. 2017 | x | / | / | x | / | / | / | / | / | Include |
| 41. | Zimmerman et al. 2017 | x | / | x | / | / | / | / | / | / | Include |
| 42. | Al Jaghsi et al. 2018 | x | / | x | / | / | / | / | / | / | Include |
| 43. | Arruda et al. 2018 | / | / | / | / | / | / | / | / | / | Include |
| 44. | Weintraub et al. 2018 | x | / | x | / | / | / | / | / | / | Include |
| 45. | Srinivasan et al. 2019 | / | / | / | / | / | / | / | / | / | Include |
| 46. | Klotz et al. 2020 | x | / | / | x | / | / | / | / | / | Include |
| 47. | Alqarni et al. 2021 | / | / | / | / | / | / | / | / | / | Include |
| 48. | Klotz et al. 2021 | x | / | / | x | / | / | / | / | / | Include |
| 49. | Ng et al. 2021 | / | / | x | / | / | / | / | / | / | Include |
| 50. | Able et al. 2022 | x | - | x | x | / | / | / | / | / | Include |
| 51. | Araujo et al. 2022 | / | / | / | / | / | / | / | / | / | Include |
| 52. | Valencia-Heredia et al. 2022 | x | / | x | / | / | / | / | / | / | Include |
| 53. | Bleiel et al. 2023 | / | / | x | / | / | / | / | / | / | Include |
| 54. | Chan et al. 2023 | / | / | / | / | / | / | - | / | / | Include |
| 55. | Gong et al. 2023 | x | / | x | / | / | / | - | / | / | Include |
| 56. | Lim et al. 2023 | / | / | / | / | / | / | / | / | / | Include |
| 57. | Viebranz et al. 2023 | x | / | / | x | / | / | / | / | / | Include |

/, Yes; x, No; -, Unclear

Appendix 4. Sensitivity analysis after excluding studies that assessed denture surfaces other than fitting surface.


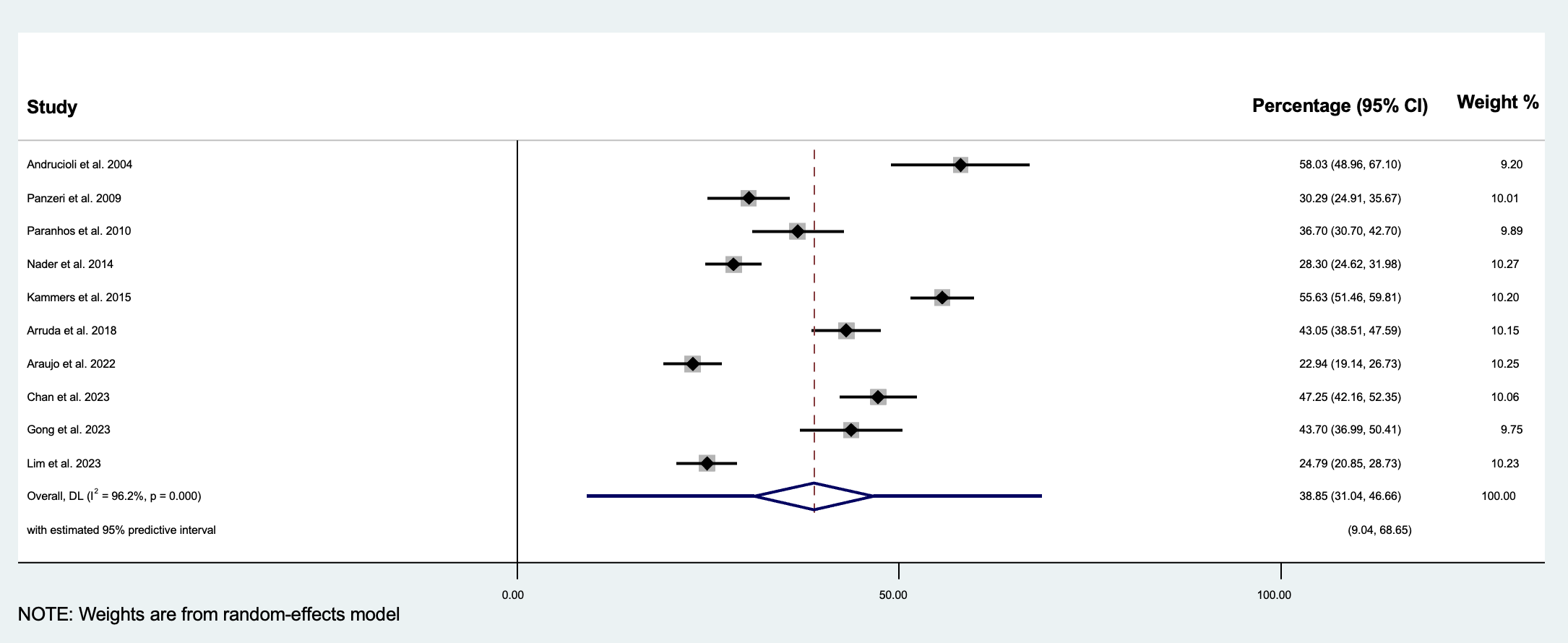

Supplement: Supplementary file 1 — Appendix 1: Search strategy. Appendix 2: Joanna Briggs Institute (JBI) critical appraisal checklist questions. Appendix 3: Quality assessment of included studies, Joanna Briggs Institute critical appraisal checklist. Appendix 4: Sensitivity analysis after excluding studies that assessed denture surfaces other than the fitting surface. [file CRE2-12-e70319-s001.docx]
